# Silicone wristbands compared with traditional polycyclic aromatic hydrocarbon exposure assessment methods

**DOI:** 10.1007/s00216-018-0992-z

**Published:** 2018-04-02

**Authors:** Holly M. Dixon, Richard P. Scott, Darrell Holmes, Lehyla Calero, Laurel D. Kincl, Katrina M. Waters, David E. Camann, Antonia M. Calafat, Julie B. Herbstman, Kim A. Anderson

**Affiliations:** 1 Food Safety and Environmental Stewardship Program, Environmental and Molecular Toxicology, Oregon State University, 1007 Agricultural and Life Sciences Building, Corvallis, OR 97331 USA; 2 Columbia Center for Children’s Environmental Health, Department of Environmental Health Sciences, Mailman School of Public Health, Columbia University, 722 West 168th Street, New York, NY 10032 USA; 3 College of Public Health and Human Sciences, Department of Environmental and Occupational Health, Oregon State University, 160 SW 26th St, Corvallis, OR 97331 USA; 4 Biological Sciences Division, Pacific Northwest National Laboratory, P.O. Box 999, Richland, WA 99352 USA; 5 Chemistry and Chemical Engineering Division, Southwest Research Institute, P.O. Drawer 28510, San Antonio, TX 78228-0510 USA; 6 Division of Laboratory Sciences, National Center for Environmental Health, Centers for Disease Control and Prevention, 1600 Clifton Road, Atlanta, GA 30333 USA

**Keywords:** Passive sampling, Active sampling, Biomonitoring, Personal monitoring, Environmental toxicology, Exposome

## Abstract

Currently there is a lack of inexpensive, easy-to-use technology to evaluate human exposure to environmental chemicals, including polycyclic aromatic hydrocarbons (PAHs). This is the first study in which silicone wristbands were deployed alongside two traditional personal PAH exposure assessment methods: active air monitoring with samplers (i.e., polyurethane foam (PUF) and filter) housed in backpacks, and biological sampling with urine. We demonstrate that wristbands worn for 48 h in a non-occupational setting recover semivolatile PAHs, and we compare levels of PAHs in wristbands to PAHs in PUFs-filters and to hydroxy-PAH (OH-PAH) biomarkers in urine. We deployed all samplers simultaneously for 48 h on 22 pregnant women in an established urban birth cohort. Each woman provided one spot urine sample at the end of the 48-h period. Wristbands recovered PAHs with similar detection frequencies to PUFs-filters. Of the 62 PAHs tested for in the 22 wristbands, 51 PAHs were detected in at least one wristband. In this cohort of pregnant women, we found more significant correlations between OH-PAHs and PAHs in wristbands than between OH-PAHs and PAHs in PUFs-filters. Only two comparisons between PAHs in PUFs-filters and OH-PAHs correlated significantly (*r*_s_ = 0.53 and *p* = 0.01; *r*_s_ = 0.44 and *p* = 0.04), whereas six comparisons between PAHs in wristbands and OH-PAHs correlated significantly (*r*_s_ = 0.44 to 0.76 and *p* = 0.04 to <0.0001). These results support the utility of wristbands as a biologically relevant exposure assessment tool which can be easily integrated into environmental health studies.

**Electronic supplementary material:**

The online version of this article (10.1007/s00216-018-0992-z) contains supplementary material, which is available to authorized users.

**Graphical abstract:**
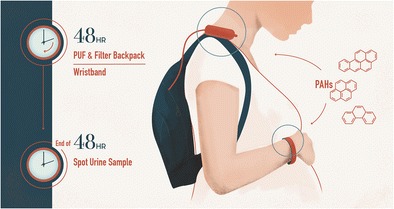
PAHs detected in samples collected from urban pregnant women

## Introduction

The assessment of an individual’s exposure to chemicals in the environment is critical to understanding if and how these exposures may affect human health. Identifying links between environmental chemical exposure and health continues to be a focus of exposure science and environmental epidemiology studies [1, 2]. Despite the importance of chemical exposure assessment, there is little information about the frequency and magnitude of personal exposures to many chemicals [3]. In addition, there is a lack of easy-to-use technology for accurate assessment of personal exposure to environmental chemicals.

To assess human exposure to environmental pollutants, researchers rely on a variety of methods, including biomarker analysis from biological matrices and active and passive sampling technologies. Researchers commonly measure biomarkers in biological samples such as urine, blood, or breast milk to assess chemical exposure [4]. Importantly, biomarker concentrations integrate all exposure routes such as inhalation, ingestion, and dermal contact [5]. Researchers can use pharmacokinetics to estimate internal exposure. However, biomarkers do not indicate the route or source of exposure [6]. Biomonitoring projects, such as the U.S. National Health and Nutrition Examination Survey (NHANES), use biomarkers in blood and urine to provide a comprehensive assessment of chemical exposures relevant to the U.S. general population [7]. It can be difficult to control for inter- and intra-individual variation when analyzing biomarker concentrations because many factors influence chemical exposure magnitude [4, 8]. Chemical toxicokinetics and exposure event timing can also influence biomarker concentrations. Biomarker analysis can be challenging when nonpersistent chemicals of interest have short biological half-lives and because biological samples need to be collected promptly after exposure [4, 6]. Yet, chemical exposures often recur and biomarkers can be a representative measure of exposure when exposure occurs on timescales that are less than a chemical’s metabolic half-life [4, 9].

Researchers often use active sampling devices, such as air-monitoring backpacks, to quantify environmental contaminants in an individual’s breathing space [10, 11]. These devices include a battery pack and pump that continuously sample air at a known flow rate during a study period. Individuals carry the device with them for the duration of the study. A polyurethane foam (PUF) cartridge collects gaseous-phase chemicals downstream of a filter that collects particle-associated chemicals [10]. However, pump noise and the requirement to carry the backpack during the study can burden some participants and influence participants’ behavior [10, 12]. Active air monitoring equipment also requires a battery supply and routine maintenance to ensure proper calibration.

Passive sampling is another established method for measuring trace levels of contaminants, and researchers often use passive samplers to detect chemicals in air and water environments [13, 14]. Organic chemicals from the environment diffuse into the lipophilic membrane of the passive sampling polymer [13]. Unbound volatile and semivolatile chemicals in the environment can then be extracted and quantified [13, 15]. Several ecological examples demonstrate that passive samplers absorb chemicals in a process similar to chemical uptake across an organism’s phospholipid membranes [14, 16]. Thus, passive samplers reflect the bioavailable fraction of lipophilic organic chemicals [14, 16]. A new application of passive sampling uses silicone wristbands to capture personal chemical exposure [17, 18]. When appropriately prepared, wristbands provide a simple method for evaluating personal exposure to select organic chemicals in the gaseous phase. Wristbands sequester a wide variety of target analytes, including polycyclic aromatic hydrocarbons (PAHs), oxygenated PAHs, flame retardants, and pesticides [15, 17–21]. Furthermore, wristbands are easy to wear [20], wristbands do not require battery power or maintenance, and the transport and stability of a wide range of chemicals in the wristbands have been evaluated [18].

In this study, we compare PAH exposure assessment methodologies. To our knowledge, this is the first time PAH concentrations in wristbands have been compared to two other existing PAH exposure assessment methods. PAHs are pervasive chemicals in the environment and exposure to certain PAHs has been associated with pathologies such as cancer, obesity, neurological issues, and respiratory distress [22–24]. Common PAH exposure sources include motor vehicle exhaust, tobacco smoke, certain stoves and heating appliances, and smoked or charbroiled foods [22, 25]. Environmental exposure can also originate from oil spills, petroleum products, and natural gas extraction [26]. PAHs are semivolatile organic compounds (SVOCs) that are present both as gaseous airborne chemicals and as chemicals adsorbed to the surfaces of airborne and settled particles [27]. Although lower molecular weight PAHs are primarily in the gaseous phase, PAHs with higher molecular weights are also present in the gaseous phase [28, 29]. PAHs in the gaseous phase are a major contributor to PAH-associated health risks [30]. Measuring PAHs in the gaseous phase is relevant when assessing personal PAH exposure.

We leveraged an ongoing and established urban birth cohort being monitored for PAH exposure by deploying wristbands alongside air-monitoring backpacks and urine sample collections. The aims of this study were threefold: (1) demonstrate that wristbands capture and recover semivolatile PAHs in 48-h deployments, (2) compare and characterize levels of PAHs in wristbands and PUFs-filters, and (3) compare and characterize levels of PAHs in wristbands with urinary concentrations of PAH biomarkers. The present exploratory study, in partnership with the Columbia Center for Children’s Environmental Health (CCCEH), demonstrates a new approach to studying PAH exposures. Paired exposure assessment studies such as this are critical to developing and integrating new technologies in exposure science and epidemiological studies.

## Materials and methods

### Study cohort

At CCCEH in New York City, 22 pregnant women obtaining prenatal care at the Farrell Community Health Center enrolled in a longitudinal epidemiologic birth cohort study. Our exploratory study leverages the Center’s ongoing and well-established birth cohort studies, which have incorporated the use of air-monitoring backpacks to measure prenatal PAH exposure during a 48-h window in the third trimester of pregnancy since 1998 [31]. CCCEH researchers also routinely collect prenatal urine samples from the mother following this 48-h period to assess PAH metabolite concentrations. We obtained informed consent from the participants in agreement with the Columbia University Institutional Review Board (IRB), the IRB of record. The involvement of the Centers for Disease Control and Prevention (CDC) laboratory did not constitute engagement in human subject research.

### Study design

Wristbands (as described in O’Connell et al. [17]) and air-monitoring backpacks (as described in Perera et al. [31]) were deployed simultaneously for 48 h. For the prenatal monitoring visit, staff instructed the participants to wear the backpack and wristband for all waking hours, from drop-off to pick-up 48 h later. If participants were sitting or sleeping, participants could remove their backpack and place it on a chair nearby. At the end of sampling, we collected all samplers and one spot urine sample. Sample collection took place between 2013 and 2015. Eighty-two percent of the population was of Dominican origin, and 23% of the population had completed 4 years of college.

### Wristband methodology

#### Preparation and deployment

We purchased 1.3 by 6.4 cm wristbands from 24hourwristbands.com (Houston, TX, USA). Prior to use, the wristbands were cleaned with five rounds of solvent exchange as described previously [17]. Briefly, the first three exchanges used a 1:1 ethyl acetate and hexane solution and the last two exchanges used a 1:1 methanol and ethyl acetate solution. The wristbands were then vacuum dried [17], individually packaged in airtight polytetrafluoroethylene (PTFE) bags (Welch Fluorocarbon, Dover, NH, USA), and mailed to CCCEH. We instructed the participants not to place personal care products, such as lotion, directly on the wristbands.

#### Cleaning and extraction

Chemical and solvent information is provided in the “Electronic supplementary material” (ESM). After deployment, field staff at CCCEH shipped the wristbands to Oregon State University (OSU) in PTFE bags. We cleaned the wristbands twice with 18 MΩ cm water and once with isopropanol to remove particles on the surface. The wristbands were immediately stored in amber jars at −20 °C until extraction. Wristbands were extracted as reported previously [17]. Briefly, we spiked each wristband with extraction surrogates (see Table S1 in the ESM) to account for extraction efficiency. We then extracted each wristband twice in 100 mL ethyl acetate at room temperature using an orbital shaker set at 60 rotations per minute and quantitatively concentrated using TurboVap^®^ closed cell evaporators (Biotage LLC, Charlotte, NC, USA). The samples were stored at −20 °C until analysis.

#### Chemical analysis

We quantitatively analyzed wristband samples for 62 PAHs with an Agilent (Santa Clara, CA, USA) 7890A gas chromatograph interfaced with an Agilent 7000 MS/MS, as described in Anderson et al. [32]. We used an Agilent Select PAH column, and each PAH in the method was calibrated with a curve of at least five points (correlations ≥0.99). Instrumental limits of detection (LOD) for the 62 PAHs, reported in Anderson et al. [32], range from 0.24 to 6.44 ng extract^−1^, with an average LOD of 0.98 ng extract^−1^. For the 20 PAHs measured in both wristband extracts and PUF-filter extracts, the average wristband LOD is 0.75 ng extract^−1^. A complete list of target PAHs are included in Table S1 (see the ESM).

### Air-monitoring backpack methodology

#### Preparation and deployment

A personal sampler (URG-2000-25A, URG, Chapel Hill, NC, USA) containing a pre-cleaned quartz microfiber filter and a pre-cleaned PUF plug (Whatman QMA, Maidstone, UK) was worn by each participant. Prior to deployment, samples were stored and prepared at Southwest Research Institute (SWRI, San Antonio, TX, USA) at −4 °C. We cut PUFs with a stainless steel die and pre-cleaned all PUFs and filters before shipping the samples to CCCEH. Prior to each deployment, we calibrated and leak tested each air-monitoring backpack as described previously [31]. During deployment, we attached the sampling head to the backpack shoulder strap in order to be close to the individual’s breathing zone. The PUF cartridge was located downstream of the filter. The filter collected particles ≤2.5 μm in diameter and the PUF collected gaseous-phase organic chemicals [31]. A second PUF was not included in the backpacks for this ongoing study because no PAH breakthrough has been found previously in the CCCEH studies [33]. The personal air-sampling pumps operated continuously for the entire sampling period at 4 L min^−1^. We instructed participants that they should not turn off the air-monitoring backpack and that they should phone field staff immediately in the event of backpack equipment failure (e.g., battery drains or pump fails).

#### PUF and filter extraction

At SWRI, we Soxhlet-extracted each PUF and filter with 6% diethyl ether in hexane for at least 16 h and concentrated to a final extract volume of 1 mL. Prior to extraction, we added extraction surrogates to each PUF and filter sample (1-methylnaphthalene-d_10_ and *p*-terphenyl-d_14_).

#### Chemical analysis

We analyzed the PUFs and filters separately to compare PAHs from the gaseous phase and from particulates. We analyzed the samples for 20 PAHs with an Agilent 6890 GC and 5973 mass-selective detector. A list of target PAHs are included in Table S2 (see the ESM). The instrumental LOD for each target PAH is 1.0 ng extract^−1^.

### Urine sample methodology

#### Collection

At the end of the 48-h sampling period, we collected a spot urine sample. The samples were kept frozen (−80 °C) at CCCEH and shipped on dry ice to the CDC for analysis.

#### OH-PAH metabolite quantification

We spiked urine samples with 100 μL of ^13^C-labeled OH-PAH internal standards, sodium acetate buffer containing β-glucuronidase/arylsulfatase enzyme, and ascorbic acid solution. After overnight enzymatic deconjugation to yield free OH-PAHs, we spiked the samples with methanol and centrifuged. We then diluted the supernatant of the sample mixture with deionized water before instrumental analysis.

We analyzed the urine samples for eight hydroxylated PAH (OH-PAH) metabolites using a Spark Holland (Emmen, Netherlands) Symbiosis online solid-phase extraction system coupled with an AB Sciex (Framingham, MA, USA) 5500/6500 high-performance liquid chromatography isotope dilution tandem mass spectrometer (HPLC-MS/MS) under the negative electrospray ionization mode [34]. A list of target OH-PAHs, including limits of detection, are included in Table S3 (see the ESM). LODs ranged from 0.007 to 0.09 ng mL^−1^ [34]. We measured creatinine using an enzymatic reaction on a Roche chemistry analyzer (Roche Hitachi, Basel, Switzerland).

### Quality control (QC)

#### Wristband QC

QC samples represent 56% of the wristband samples analyzed. We collected blank wristband samples during wristband conditioning, traveling, and cleaning. We collected solvent extraction blanks by performing the extraction process without wristbands. All blank QCs were below the LOD for 56 of the 62 PAHs. We averaged and subtracted any detected concentrations in the blanks from sample concentrations.

Average surrogate recoveries ranged from 56% to 93%, with an average recovery of 78%. Instrument concentrations were all surrogate-corrected, and all instrument blanks were below the LOD for all PAHs. During sample analysis, we analyzed instrument blanks and calibration verifications at the beginning and end of each set of wristband samples. All continuing calibration verifications were verified at ±20% of the true value for >80% of the PAHs. We analyzed continuing calibration verifications approximately every 10 samples and/or at the end of the sample set. If a closing verification did not meet the criteria, we verified the standards and re-ran the samples. Prior to wristband deployment, we extracted and analyzed two wristbands from each batch of conditioned wristbands via GC-MS with a 500 ng internal standard (perylene-d_12_) and, per our data quality objectives (DQOs), made sure there were less than four discrete peaks over 15 times the response of our internal standard. We also verified wristband color and polymer elasticity to match DQOs.

#### Air-monitoring backpack QC

After the study, each backpack underwent a quality control sampling check, factoring in compliance metrics such as duration of sampling time and air flow. Extraction surrogate recoveries for 1-methylnaphthalene-d_10_ ranged from 86% to 111%, and recoveries of *p*-terphenyl-d_14_ ranged from 100% to 201%. We also prepared and analyzed two matrix blanks (one PUF and one filter) and two matrix spikes (one PUF and one filter) of all targeted individual PAHs. Naphthalene, 2-methylnaphthalene, 1-methylnaphthalene, and phenanthrene were present in concentrations above the LOD in the PUF or filter matrix blanks. PAH concentrations in matrix blanks were subtracted from PAH concentrations in the samples.

#### Urine QC

The CDC’s QC process is described in Wang et al. [34]. Each analytical run of samples included high- and low-concentration QC materials and reagent blanks to assure the accuracy and reliability of the data. We prepared two levels of QC materials by pooling urine from smokers and nonsmokers and by fortifying the QC concentrations with native target compounds to encompass the ranges described for the U.S. general population [7]. All QC materials were stored in 4-mL amber glass vials at −70 °C until used. Additional details pertaining to the CDC’s QC process are included in the ESM.

### Data analysis

PAH concentrations are reported for wristbands worn on the wrist (*n* = 22) as ng wristband^−1^, and for PUFs and filters as ng PUF^−1^ and ng filter^−1^. For all analyses, we assigned concentrations below the LOD a value equal to one-half the LOD. For most analyses, we focus on the 20 PAHs measured in the wristbands, PUFs, and filters. We analyzed the PUFs and filters separately, but we also summed the PUF and filter sample from each participant to provide us with an additional metric, PUF-filter. We applied a creatinine correction to OH-PAH concentrations (reported in ng g^−1^ creatinine) to adjust for urine dilution.

We conducted statistical analyses using the statistical software R, version 3.1.1, and JMP Pro, version 12.0.1. We used a nonparametric analysis because we did not assume the data from 22 participants to be normally distributed. Therefore, we calculated nonparametric, Spearman’s rank-order correlations (*r*_s_) to evaluate the relationships between PAH concentrations in the PUFs-filters and wristbands and OH-PAH urinary concentrations. The correlation results do not change if we use PAH air concentrations (e.g., ng/m^3^) instead of ng PUF^−1^ and ng PUF-filter^−1^. We considered an *r*_s_ coefficient of 0.20–0.39 to be weak, 0.40–0.59 to be moderate, 0.60–0.79 to be strong, and ≥0.81 to be very strong (adapted from [35, 36]). We did not exclude any values that were below the LOD from the reported correlations. Statistical significance was set at *α* = 0.05 for all analyses.

## Results

### PAHs in wristbands

Of the 62 PAHs tested in the 22 wristbands, 51 were detected in at least one wristband (Table 1). The median PAH concentrations from all 22 wristbands were highest for phenanthrene (228 ng wristband^−1^), naphthalene (87 ng wristband^−1^), and fluorene (74 ng wristband^−1^).

**Table 1:** PAH detection frequencies in wristbands, PUFs, and filters. Of the 62 PAHs, the average LOD for wristband extracts is 0.98 ng extract^−1^. The LOD for PUF and filter extracts is 1.0 ng extract^−1^

	Of 62 PAHs tested		Of 20 PAHs tested	
	Number of PAHs detected in ≥1 sample	Number of PAHs detected in >50% of samples	Number of PAHs detected in ≥1 sample	Number of PAHs detected in >50% of samples
Wristband	51	31	20	17
PUF			18	12
Filter			18	11
PUF-filter			20	18

### PAHs in wristbands and PUFs-filters

All of the 20 PAHs quantified in both the wristbands and PUFs-filters were detected in at least one wristband and in at least one combined PUF-filter (Table 1). Of the 42 PAHs tested only in the wristbands and not in the PUFs-filters, 14 were detected in over 50% of wristbands and 31 were found in at least one wristband.

The median PAH concentrations from all PUFs were highest for phenanthrene (440 ng PUF^−1^), fluorene (228 ng PUF^−1^), and naphthalene (207 ng PUF^−1^). The median PAH concentrations from all 22 filters were highest for benzo[*ghi*]perylene (5 ng filter^−1^), benzo[*b*]fluoranthene (4 ng filter^−1^), and indeno[1,2,3-*cd*]pyrene (3 ng filter^−1^).

**Fig. 1a–c:**
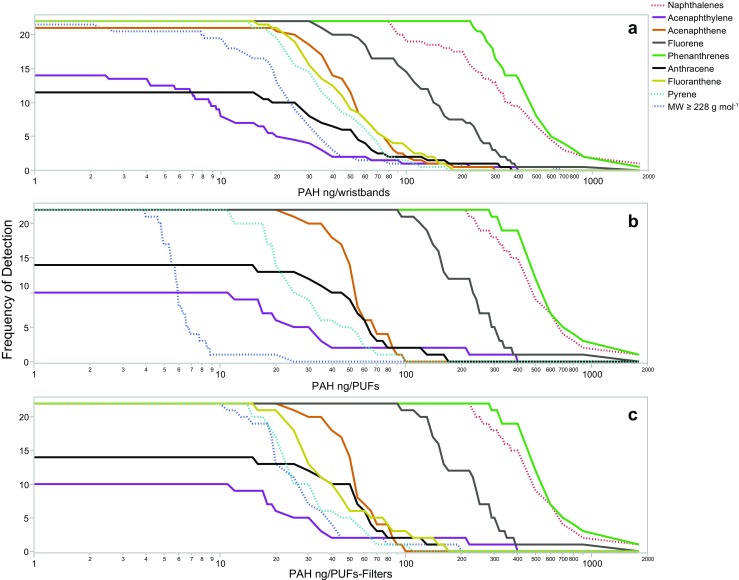
PAH frequency of detection and concentration in 22 wristbands (**a**), PUFs (**b**), and PUFs and filters combined (**c**). The 20 PAHs measured in both the wristbands and PUFs-filters are represented in this figure. The *red dotted line* *labeled “naphthalenes”* includes the sum of three PAHs (naphthalene, 2-methylnaphthalene, and 1-methylnaphthalene). The *green solid line* *labeled “phenanthrenes”* includes the sum of three PAHs (phenanthrene, 2-methylphenanthrene, and 1-methylphenanthrene). The *dark blue dotted line labeled “MW ≥ 228 g mol*^*−1*^*″* includes the sum of the eight PAHs in this group of 20 PAHs with a molecular weight ≥ 228 g mol^−1^ (benz[*a*]anthracene, chrysene/isochrysene, benzo[*b*]fluoranthene, benzo[*k*]fluoranthene, benzo[*a*]pyrene, indeno[1,2,3-*cd*]pyrene, dibenz[*a*,*h*]anthracene, and benzo[*ghi*]perylene). PAH concentrations are represented on a log scale. For these 20 PAHs, the average LOD for wristband extracts is 0.75 ng extract^−1^ and that for PUF and filter extracts is 1.0 ng extract^−1^

In the three different media (wristbands, PUFs, and PUFs-filters), naphthalene, fluorene, phenanthrene, and their related alkylated compounds were detected in all 22 samples and at the highest concentrations (Fig. 1a–c). In Table 2, the number of detections of each PAH in the 22 wristband, PUF, and filter samples are listed. Acenaphthene, fluoranthene, and pyrene had similar detection frequencies and concentrations in the three different media (Fig. 1a–c). The frequency of detection for acenaphthylene was higher in wristbands than in either PUFs or PUFs-filters combined. The frequency of detection for anthracene was higher in either PUFs alone or PUFs-filters combined than in wristbands. Because of the presence of additional chemicals at the same elution times as acenaphthylene and anthracene, some PUF samples in this study may have had elevated concentrations of anthracene and had higher detection limits for acenaphthylene (data not shown), which may explain the frequency of detection trends for these two PAHs.

**Table 2:** Number of detections of each PAH in 22 wristband, PUF, and filter samples. For these 20 PAHs, the average LOD for wristband extracts is 0.75 ng extract^−1^ and that for the PUF and filter extracts is 1.0 ng extract^−1^

PAH	Detections out of 22 samples		
	Wristband *n*	PUF *n*	Filter *n*
Naphthalene	22	22	13
2-Methylnaphthalene	22	22	13
1-Methylnaphthalene	22	22	2
Acenaphthylene	18	10	1
Acenaphthene	20	22	0
Fluorene	22	22	1
Phenanthrene	22	22	18
Anthracene	10	14	0
2-Methylphenanthrene	22	22	1
1-Methylphenanthrene	15	22	1
Fluoranthene	22	22	17
Pyrene	22	22	14
Benz[*a*]anthracene	17	21	18
Chrysene/isochrysene	17	10	11
Benzo[*b*]fluoranthene	21	2	18
Benzo[*k*]fluoranthene	17	1	12
Benzo[*a*]pyrene	13	2	17
Indeno[1,2,3-*cd*]pyrene	2	2	21
Dibenzo[*a*,*h*]pyrene	1	0	1
Benzo[*ghi*]perylene	16	0	21

The greatest difference between the PAHs detected in the PUFs compared to the PUFs-filters was for the eight PAHs with molecular weights greater than or equal to the molecular weight of benz[*a*]anthracene (228.29 g mol^−1^). In Fig. 1, these eight PAHs are summed. Both the frequencies of detection and concentrations of these eight PAHs were visually comparable between the wristbands (Fig. 1a) and PUFs-filters combined (Fig. 1c).

Spearman’s correlation coefficients were calculated to compare wristband and PUF-filter concentrations of PAHs detected in >50% of samples (Table 3). The Spearman’s correlation coefficients for the PUF and wristband comparisons indicated moderate or better correlations for seven of the 11 PAHs (Table 3). There were strong correlations between the PAH concentrations in wristbands and those in PUFs for three of the relatively low molecular weight PAHs (naphthalene, 128.17 g mol^−1^; acenaphthene, 154.20 g mol^−1^; and fluorene, 166.22 g mol^−1^). There were moderate correlations for 2-methylnaphthalene, 1-methylnaphthalene, phenanthrene, and fluoranthene.

**Table 3:** Correlation table for 20 PAHs analyzed in air-monitoring backpacks (PUFs and filters) and wristbands

PAH	Wristband PAH and PUF PAH		Wristband PAH and PUF-filter PAH	
	*r*_s_	*p*-value	*r*_s_	*p*-value
Naphthalene	**0.71**	0.0002*	**0.71**	0.0002*
2-Methylnaphthalene	**0.47**	0.03*	**0.47**	0.03*
1-Methylnaphthalene	**0.49**	0.02*	**0.49**	0.02*
Acenaphthylene	a	a	a	a
Acenaphthene	**0.69**	0.0004*	**0.69**	0.0004*
Fluorene	**0.71**	0.0002*	**0.71**	0.0002*
Phenanthrene	**0.54**	0.009*	**0.54**	0.009*
Anthracene	b	b	b	b
2-Methylphenanthrene	0.15	0.50	0.14	0.53
1-Methylphenanthrene	0.41	0.06	**0.43**	0.05*
Fluoranthene	**0.56**	0.007*	**0.54**	0.009*
Pyrene	0.26	0.24	0.28	0.20
Benz[*a*]anthracene	−0.03	0.90	0.03	0.89
Chrysene/isochrysene	a	a	0.09	0.69
Benzo[*b*]fluoranthene	a	a	0.23	0.29
Benzo[*k*]fluoranthene	a	a	0.18	0.43
Benzo[*a*]pyrene	a	a	0.15	0.52
Indeno[1,2,3-*cd*]pyrene	c	c	b	b
Dibenz[*a*,*h*]anthracene	c	c	c	c
Benzo[*ghi*]perylene	a	a	0.33	0.13

^a^ >50% detections in wristbands and <50% detections in PUFs and filters

^b^ >50% detections in PUFs and filters and <50% detections in wristbands

^c^ <50% detections in wristbands and PUFs and filters

* and **bold type** indicate *α* < 0.05

The Spearman’s correlation coefficients did not substantially change whether the PUFs were analyzed alone or added to the filter concentrations. The Spearman’s correlation coefficients for the PUF-filter and wristband comparisons indicated moderate or better correlations for eight of the 16 PAHs (Table 3). Even when the filter was included, the five comparisons for the benz[*a*]anthracene, chrysene/isochrysene, benzo[*b*]fluoranthene, benzo[*k*]fluoranthene, benzo[*a*]pyrene, and benzo[*ghi*]perylene concentrations demonstrated little to no correlation between wristbands and PUFs-filters.

### PAHs in wristbands and PAH metabolites in urine

All eight OH-PAHs were detected in all urine samples. For naphthalene, fluorene, and phenanthrene, the predominant metabolites were 2-OH-naphthalene, 2-OH-fluorene, and 1-OH-phenanthrene, respectively (Table S4 in the ESM).

Spearman’s correlation coefficients were calculated to compare the PAH concentrations in the wristbands, PUFs, and PUFs-filters with the OH-PAH concentrations in urine (Table 4). Three additional correlation coefficients were assessed: the two metabolites of naphthalene were summed, as were the two fluorene metabolites and the three phenanthrene metabolites. PAH concentrations in PUFs and OH-PAH concentrations in urine were moderately correlated in two of the 11 comparisons (naphthalene and 1-OH-naphthalene, fluorene and 2-OH-fluorene; Table 4). All correlation inferences remained the same when the PUFs and filters were combined. PAH concentrations in the wristband and OH-PAH concentrations in the urine were positively correlated in six of the 11 comparisons. Wristbands moderately or strongly correlated with the 1-OH-metabolites in urine, including 1-OH-naphthalene, 1-OH-phenanthrene, and 1-OH-pyrene. Two of the 11 comparisons resulted in strong, significant correlations between the wristband and urine samples (phenanthrene and 1-OH-phenanthrene, *r*_s_ = 0.76, *p* < 0.0001; pyrene and 1-OH-pyrene, *r*_s_ = 0.66, *p* = 0.0009; Table 4; Fig. S1 in the ESM).

**Table 4:** Correlation table for creatinine-corrected OH-PAHs in urine and PAHs in backpacks (PUFs and filters) and wristbands

PAH	PAH metabolite	Urine PAH metabolite and PUF PAH		Urine PAH metabolite & PUF-filter PAH		Urine PAH metabolite & wristband PAH	
		*r*_s_	*p*-value	*r*_s_	*p*-value	*r*_s_	*p*-value
Naphthalene	1-OH-naphthalene	**0.53**	0.01*	**0.53**	0.01*	**0.48**	0.02*
	2-OH-naphthalene	0.27	0.23	0.27	0.23	**0.44**	0.04*
	ΣOH-naphthalene^a^	0.35	0.11	0.35	0.11	**0.47**	0.03*
Fluorene	2-OH-fluorene	**0.44**	0.04*	**0.44**	0.04*	0.33	0.13
	3-OH-fluorene	0.08	0.72	0.08	0.72	0.14	0.52
	ΣOH-fluorene^b^	0.33	0.13	0.33	0.13	0.27	0.22
Phenanthrene	1-OH-phenanthrene	0.18	0.41	0.18	0.41	**0.76**	<0.0001*
	2- and 3-OH-phenanthrene	0.22	0.33	0.22	0.33	0.37	0.09
	4-OH-phenanthrene	0.23	0.30	0.23	0.30	0.18	0.42
	ΣOH-phenanthrene^c^	0.20	0.38	0.20	0.38	**0.64**	0.002*
Pyrene	1-OH-pyrene	0.11	0.63	0.12	0.59	**0.66**	0.0009*

^a^ Sum of 1-OH-naphthalene and 2-OH-naphthalene concentrations

^b^ Sum of 2-OH-fluorene and 3-OH-fluorene concentrations

^c^ Sum of 1-OH-phenanthrene, 2- and 3-phenanthrene, and 4-OH-phenanthrene concentrations

* and **bold type** indicates *α* < 0.05

## Discussion

### Captured and Recovered PAHs

We demonstrated for the first time that wristbands capture and recover PAHs in a 48-h non-occupational exposure period. Wristbands recovered PAHs with similar frequencies of detection and concentrations to PUFs and filters in this study. It is advantageous to develop additional PAH exposure assessment tools, such as the wristband, to improve public health research pertaining to PAH exposure.

This study leveraged PUF and filter samples already collected and analyzed for an ongoing urban birth cohort, which explains why the PUFs-filters were tested for 20 PAHs while the wristbands were tested for 62 PAHs. However, even though PAHs are a commonly studied chemical class, by only analyzing the PUFs and filters for 20 PAHs, researchers exclude important exposure information. For instance, by analyzing the wristbands in this study for an additional 42 PAHs, we detected an additional 31 PAHs. Low-concentration PAHs are important exposures to report in addition to high-concentration PAHs as they might contribute to adverse health effects depending on their toxic potential. For example, benzo[*c*]fluorene, a PAH with a relative potency factor of 20 for cancer risk [37], was detected in seven of the 22 wristbands. PAH relative potency factors are assigned relative to the potency of benzo[*a*]pyrene, an index compound known to be carcinogenic. These results demonstrate the importance of analyzing a large number of chemicals simultaneously in an appropriate matrix.

### Wristbands compared to other PAH exposure assessment methods

Wristbands have been used in several research studies previously [15, 17–21], but PAH concentrations in wristbands have never been compared with PAH concentrations from active PUF and filter samples and OH-PAH concentrations from urine. Hammel et al. compared the use of wristbands, hand wipes, and urine for assessing exposure to organophosphate flame retardants (OPFRs) [19]. Significant correlations were found between OPFRs in the wristbands and corresponding urinary metabolites, and Hammel et al. suggested that wristbands may be an improved OPFR exposure metric over hand wipes [19].

PAH metabolites in urine and PAHs in air samplers have been quantified together in other non-occupational studies [11, 38–41]. Most of these studies found little to no association between PAHs in air and corresponding OH-PAHs in urine [11], with the exception of the study by Li et al. where moderate to strong correlations were found between PUF and filter PAH concentrations and urinary OH-PAH concentrations for naphthalene and fluorene [41]. In that study, when the PUF and filter PAHs measured did not associate well with urine PAH metabolites, dietary PAH exposure was hypothesized to explain the lack of correlations, especially for PAHs with three or more rings such as pyrene [41]. With this dietary hypothesis, we would expect few correlations between wristbands and urine for PAHs of greater molecular weight than fluorene because wristbands do not incorporate dietary exposures. Yet, in this study, there were strong, significant correlations for PAH and OH-PAH comparisons between wristbands and urine samples for phenanthrene and pyrene. These correlation patterns could have been the result of wristbands incorporating dermal exposure, wristbands being in close proximity to PAH point sources, and/or wristbands selectively capturing the bioavailable PAH fraction.

#### Dermal exposure

Ingestion and inhalation are often reported as the dominant PAH exposure routes for the general population [41, 42]. Yet, wristbands may capture dermal PAH exposure [15], an exposure route not well researched in non-occupational studies. Estimates indicate that SVOC uptake by skin absorption can be larger than previously thought—potentially equal to, or in some cases exceeding, SVOC uptake by inhalation [43]. While PUF and filters capture PAHs in the air of a person’s breathing zone [10], wristbands may sequester PAHs in contact with a person’s skin, which can include PAHs from air, water, and/or personal care products.

Particulates on wristband surfaces are not a source of PAHs in this study because the wristbands were cleaned before analysis to remove surface particulates and superficial fouling [20]. PAH correlation patterns were unchanged when the filter was added to the PUF, which suggests that particulates in the participants’ environments were not a strong source of PAHs. Additionally, concentrations of heavier molecular weight PAHs (≥228 g mol^−1^) detected in the filters did not correlate well with the concentrations detected in the wristbands, reaffirming that particulates are not a source of PAHs in wristbands. Regardless, dermal exposure to gaseous-phase PAHs in non-occupational populations may be larger than previously thought; this warrants further investigation and inclusion in exposure assessments.

#### Proximity to PAH point sources

Wristbands worn on the wrist may be in closer proximity to localized PAH exposure sources, such as broiling food, than PUFs-filters, which may partly explain the strong correlations between PAHs in wristbands and OH-PAHs in urine. PUFs-filters that are located near a person’s breathing zone or placed on a nearby chair while the participant is sitting or sleeping may not capture PAH exposure from point sources as fully as a sampler worn on the wrist.

#### Bioavailable PAH fraction

The chemical profile sequestered by silicone passive samplers may be more representative of the bioavailable fraction of PAHs from the environment than the profile sequestered by active air samplers, and this fraction correlates better with urinary biomarker concentrations than with the PUF and filter. There is a lack of literature examining the similarities and differences between PUF and silicone polymers and how these polymers differ when used for passive or active sampling. However, passive samplers absorb lipophilic organic contaminants via simple diffusion from the environment [13]. The diffusion process is similar to chemical uptake across a phospholipid membrane into an organism [14, 16, 44]. Paulik et al. demonstrated that passive samplers can accurately estimate PAH contamination in crayfish [14]. The passive sampling silicone polymer may better sequester the bioavailable fraction of PAHs than active air samplers, as demonstrated by strong correlations between wristband PAHs and urine OH-PAHs. The PUF in the active air sampler may not sequester the bioavailable fraction as well as the silicone, and this could explain the two weaker correlations between PAH and OH-PAH pairs sampled by the PUF-filter and urine.

### Additional considerations

Although wristbands may capture more exposure information about an individual’s external environment than PAHs in air, such as PAHs in contact with a person’s skin, this can also be a limitation. Current human health chemical risk assessments use defined exposure routes, such as inhalation, and it is difficult at this time to use wristband concentrations in these specific risk assessment calculations. In future work, performance reference compounds, routinely used with passive samplers [13, 27], can be applied to the wristbands to calculate PAH air concentrations and to assist in the delineation of exposure routes. A subset of paired wristband and PUF-filter PAH concentrations from this study was used in Anderson et al. to develop the first estimates of wristband–air partitioning coefficients, which will aid in calculating future environmental air concentrations from wristbands [18]. Additionally, if dermal exposure is not desired, the wristband can also be worn as a lapel, as demonstrated in O’Connell et al. [17], or on top of a nonpermeable wristband cuff. The unique ability to combine dermal and inhalation exposure can also be an asset in exposure assessment because there is a current focus within the field of exposure science on measuring the totality of personal chemical exposures [1, 3].

Interference compounds were present in some PUF samples. This may have led to higher concentrations of anthracene and higher detection limits for acenaphthylene, which could influence the detection frequencies and correlation coefficients between PUFs and wristbands (Fig. 1, Tables 2 and 3) for these two PAHs. Additional research is needed to confirm the PUF-related anthracene and acenaphthylene trends found in this study.

We collected a spot urine sample in this study. We determined that collecting a 48-h urine void would be too burdensome on participants, and research on personal exposure to PAHs regularly includes analyses of spot urine samples [45]. In our study, the wristband and PUF-filter reflect exposures from the entire 48-h deployment period, but the urine most likely represents a shorter window of PAH exposure. PAHs metabolize quickly [41] and do not reflect exposure beyond a few hours from urine collection [46]. However, strong, significant correlations were seen between wristband PAH concentrations and urine concentrations, suggesting that one-time urine samples captured a PAH exposure snapshot similar to the one collected by wristbands. In addition, PAH exposure is recurring and it has been demonstrated that spot urine samples can be a representative measure of exposure when the exposure is chronic and occurring on timescales of less than the compound’s metabolic half-life [4, 9].

Although OH-PAHs are commonly used as biomarkers of human exposure to PAHs [45], OH-PAHs are not unique to human metabolism. PAHs can undergo hydroxyl oxidation or photochemical transformation to form OH-PAHs in ice, water, and the atmosphere [47, 48]. OH-PAH concentrations have been reported in PM_2.5_ aerosols from Nanjing, China [49] and PM_10_ aerosols from Madrid, Spain [48]. It is highly likely that people are exposed to OH-PAHs in their everyday environment. Therefore, not all OH-PAHs in the urine may be the direct result of parent PAH metabolism in the body. Interestingly, researchers could directly quantify OH-PAHs in wristbands themselves to get an estimate of external OH-PAH exposure, which may inform future OH-PAH biomonitoring studies.

## Conclusions

Wristbands captured and recovered PAHs in a 48-h time period when worn by pregnant women in New York City. Wristbands are a candidate technology to include in environmental health studies in a similar manner to air-monitoring backpacks and urine samples. Acknowledging the small sample size in this pilot study, there were three times more positive correlations between PAH and OH-PAH pairs in wristbands and urine samples than there were between PUFs-filters and urine samples. Phenanthrene and pyrene in wristbands strongly correlated with 1-OH-phenanthrene and 1-OH-pyrene in urine, respectively. The correlation patterns from the wristband, PUF-filter, and urine comparisons could be the result of wristbands incorporating both dermal and gaseous-phase PAH exposure, wristbands being in close proximity to PAH point sources, and/or wristbands more selectively capturing the bioavailable PAH fraction. Additional investigation of these factors will help researchers to better understand personal exposure to environmental chemicals. Overall, wristbands are an easy-to-use and effective PAH external exposure assessment tool to integrate into exposure science and epidemiological studies.

## Electronic supplementary material

ESM 1 (PDF 286 kb)
