# Supplementary material for: Silicone wristbands compared with traditional polycyclic aromatic hydrocarbon exposure assessment methods
Source: Anal Bioanal Chem. 2018 Apr 2;410(13):3059–71. doi: 10.1007/s00216-018-0992-z (PMC5910488; doi:10.1007/s00216-018-0992-z)
Supplement: Supplementary file 1 — (PDF 286 kb) [file 216_2018_992_MOESM1_ESM.pdf]

Analytical and Bioanalytical Chemistry

Electronic Supplementary Material

Silicone wristbands compared with traditional polycyclic aromatic hydrocarbon exposure assessment methods

Holly M. Dixon, Richard P. Scott, Darrell Holmes, Lehyla Calero, Laurel D. Kincl, Katrina M. Waters, David E. Camann, Antonia M. Calafat, Julie B. Herbstman, Kim A. Anderson

Table of Contents

Page	Content
S3	Chemical Information for Wristband Methodology
S3	Additional CDC Quality Control Details
S4	**Table S1** PAH surrogates and target compounds in wristband analysis method.
S5	**Table S2** PAH surrogates and target compounds in PUF and filter analysis method.
S6	**Table S3** Hydroxy-PAH target compounds and LODs in urine analysis method.
S6	**Table S4** Urinary OH-PAH metabolite ratios for naphthalene, fluorene, and phenanthrene.
S7	**Fig. S1** Phenanthrene and pyrene correlation scatterplot for PAH and OH-PAH concentrations in the wristbands, backpacks, and urine samples.

Chemical Information for Wristband Methodology

We purchased PAH standards from Sigma Aldrich (St. Louis, MO), Accustandard (New Haven, CT), and Chiron (Trondheim, Norway). All solvents were Optima-grade or equivalent (Fisher Scientific, Pittsburgh, PA). All of the glassware and tools used were baked for 12 hours at 450°C and/or solvent-rinsed before use. Eighteen MΩ*cm water used to clean silicone was filtered through a D7389 purifier (Barnstead International, Dubuque, IA).

Additional CDC Quality Control Details

The CDC laboratory is certified by the Health Care Financing Administration to comply with the requirements set forth in the Clinical Laboratory Improvement Act of 1988 (CLIA '88) and is recertified every two years. At CDC, analytical measurements are conducted following strict quality control/quality assurance CLIA guidelines, and include participation in external quality assessment schemes to continuously demonstrate the accuracy and precision of CDC's analytical methods.

Table S1 PAH surrogates and target compounds in wristband analysis method

PAH	Molecular Weight (g mol^{-1})	Target, Internal Standard (IS), or Surrogate	PAH	Molecular Weight (g mol^{-1})	Target, Internal Standard (IS), or Surrogate
Perylene-d12	264.34	IS	Benzo[a]fluorene	216.23	Target
Naphthalene-d8	136.22	Surrogate	Benzo[b]fluorene	216.23	Target
Acenaphthylene-d8	160.25	Surrogate	Benzo[c]fluorene	216.23	Target
Phenanthrene-d10	188.30	Surrogate	1-Methylpyrene	216.28	Target
Fluoranthene-d10	212.31	Surrogate	Benz[a]anthracene	228.29	Target
Chrysene-d12	240.36	Surrogate	Cyclopenta[c,d]pyrene	229.27	Target
Benzo[a]pyrene-d12	264.39	Surrogate	Triphenylene	228.29	Target
Benzo[ghi]perylene-d12	288.41	Surrogate	Chrysene	228.28	Target
Naphthalene	128.17	Target	6-Methylchrysene	242.31	Target
2-Methylnaphthalene	142.20	Target	5-Methylchrysene	242.31	Target
1-Methylnaphthalene	142.20	Target	Benzo[b]fluoranthene	252.30	Target
2-Ethylnaphthalene	156.09	Target	7,12-Dimethylbenz[a]anthracene	256.34	Target
2,6-Dimethylnaphthalene	156.22	Target	Benzo[k]fluoranthene	252.30	Target
1,6-Dimethylnaphthalene	156.22	Target	Benzo[j]fluoranthene	252.30	Target
1,4-Dimethylnaphthalene	156.22	Target	Benz[j]&[e]aceanthrylene	256.31	Target
1,5-Dimethylnaphthalene	156.22	Target	Benzo[e]pyrene	252.30	Target
1,2-Dimethylnaphthalene	156.22	Target	Benzo[a]pyrene	252.30	Target
1,8-Dimethylnaphthalene	156.22	Target	Indeno[1,2,3-c,d]pyrene	276.33	Target
2,6-Diethylnaphthalene	184.27	Target	Dibenzo[a,h]pyrene	278.35	Target
Acenaphthylene	152.19	Target	Picene	278.35	Target
Acenaphthene	154.20	Target	Benzo[ghi]perylene	276.33	Target
Fluorene	166.22	Target	Anthanthrene	276.33	Target
Dibenzothiophene	184.26	Target	Naphtho[1,2-b]fluoranthene	302.36	Target
Phenanthrene	178.23	Target	Naphtho[2,3-j]fluoranthene	302.36	Target
Anthracene	178.23	Target	Dibenzo[a,e]fluoranthene	302.37	Target
2-Methylphenanthrene	192.25	Target	Dibenzo[a,l]pyrene	302.37	Target
2-Methylanthracene	192.25	Target	Naphtho[2,3-k]fluoranthene	302.37	Target
1-Methylphenanthrene	192.25	Target	Naphtho[2,3-e]pyrene	302.37	Target
9-Methylanthracene	192.25	Target	Dibenzo[a,e]pyrene	302.37	Target
3,6-Dimethylphenanthrene	206.28	Target	Coronene	300.35	Target
2,3-Dimethylanthracene	206.28	Target	Dibenzo[e,l]pyrene	302.36	Target
Fluoranthene	202.26	Target	Naphtho[2,3-a]pyrene	302.36	Target
9,10-Dimethylanthracene	206.28	Target	Benzo[b]perylene	302.36	Target
Pyrene	202.25	Target	Dibenzo[a,i]pyrene	302.36	Target
Retene	234.33	Target	Benzo[ghi]perylene	302.36	Target

Table S2 PAH surrogates and target compounds in PUF and filter analysis method

PAH	Molecular Weight (g mol^{-1})	Target, Internal Standard (IS), or Surrogate
Naphthalene-d8	136.22	IS
Acenaphthene-d10	160.25	IS
Phenanthrene-d10	188.30	IS
Chrysene-d12	240.36	IS
Perylene-d12	264.34	IS
1-Methylnaphthalene-d10	152.26	Surrogate
p-Terphenyl-d14	244.40	Surrogate
Naphthalene	128.17	Target
2-Methylnaphthalene	142.20	Target
1-Methylnaphthalene	142.20	Target
Acenaphthylene	152.19	Target
Acenaphthene	154.20	Target
Fluorene	166.22	Target
Phenanthrene	178.23	Target
Anthracene	178.23	Target
2-Methylphenanthrene	192.25	Target
1-Methylphenanthrene	192.25	Target
Fluoranthene	202.26	Target
Pyrene	202.25	Target
Benz[a]anthracene	228.29	Target
Chrysene/Iso-chrysene	228.28	Target
Benzo[b]fluoranthene	252.30	Target
Benzo[k]fluoranthene	252.30	Target
Benzo[a]pyrene	252.30	Target
Indeno[1,2,3-c,d]pyrene	276.33	Target
Dibenzo[a,h]pyrene	278.35	Target
Benzo[ghi]perylene	302.36	Target

Table S3 Hydroxy-PAH target compounds and LODs in urine analysis method

Target Hydroxy-PAH (OH-PAH)	Limit of Detection (LOD) ng/mL
1-OH-naphthalene	0.06
2-OH-naphthalene	0.09
2-OH-fluorene	0.008
3-OH-fluorene	0.008
1-OH-phenanthrene	0.009
2-OH- & 3-OH-phenanthrene	0.01
4-OH-phenanthrene	0.007
1-OH-pyrene	0.07

Table S4 Urinary OH-PAH metabolite ratios for naphthalene, fluorene, and phenanthrene. The metabolite with the lowest concentration has a value of one in each ratio

Individual	1-OH-naphthalene	:	2-OH-naphthalene	2-OH-fluorene	:	3-OH-fluorene	1-OH-phenanthrene	:	2- & 3-OH-phenanthrene	:	4-OH-phenanthrene
1	1.0	:	3.6	4.1	:	1.0	7.2	:	3.3	:	1.0
2	1.0	:	12.8	5.1	:	1.0	1.3	:	2.7	:	1.0
3	1.0	:	5.0	3.2	:	1.0	5.1	:	3.8	:	1.0
4	1.0	:	1.2	2.0	:	1.0	7.0	:	4.3	:	1.0
5	1.0	:	3.2	3.1	:	1.0	4.3	:	4.2	:	1.0
6	1.0	:	2.4	3.1	:	1.0	4.6	:	4.1	:	1.0
7	1.0	:	5.3	4.0	:	1.0	6.6	:	3.7	:	1.0
8	1.0	:	12.9	2.5	:	1.0	2.2	:	3.3	:	1.0
9	1.0	:	66.9	1.9	:	1.0	1.2	:	1.9	:	1.0
10	1.0	:	9.9	5.8	:	1.0	1.7	:	3.4	:	1.0
11	1.0	:	8.5	3.8	:	1.0	5.4	:	3.3	:	1.0
12	1.0	:	4.9	2.8	:	1.0	6.1	:	2.4	:	1.0
13	1.0	:	19.8	4.0	:	1.0	5.0	:	3.5	:	1.0
14	1.0	:	3.0	5.0	:	1.0	2.8	:	2.6	:	1.0
15	1.0	:	1.7	3.6	:	1.0	7.6	:	3.7	:	1.0
16	1.0	:	6.4	4.7	:	1.0	7.6	:	3.4	:	1.0
17	1.0	:	4.1	3.2	:	1.0	3.2	:	3.4	:	1.0
18	1.0	:	5.1	2.2	:	1.0	3.3	:	2.9	:	1.0
19	1.0	:	21.0	4.2	:	1.0	1.8	:	3.0	:	1.0
20	1.0	:	2.3	4.5	:	1.0	6.4	:	3.1	:	1.0
21	1.0	:	6.8	5.1	:	1.0	9.4	:	6.0	:	1.0
22	1.0	:	17.0	4.2	:	1.0	6.7	:	3.2	:	1.0
**Range**	1.0	:	1.2 - 66.9	1.9 - 5.8	:	1.0	1.2 - 9.4	:	1.9 - 6.0	:	1.0
**Average**	1.0	:	10.18	3.7	:	1.0	4.8	:	3.4	:	1.0

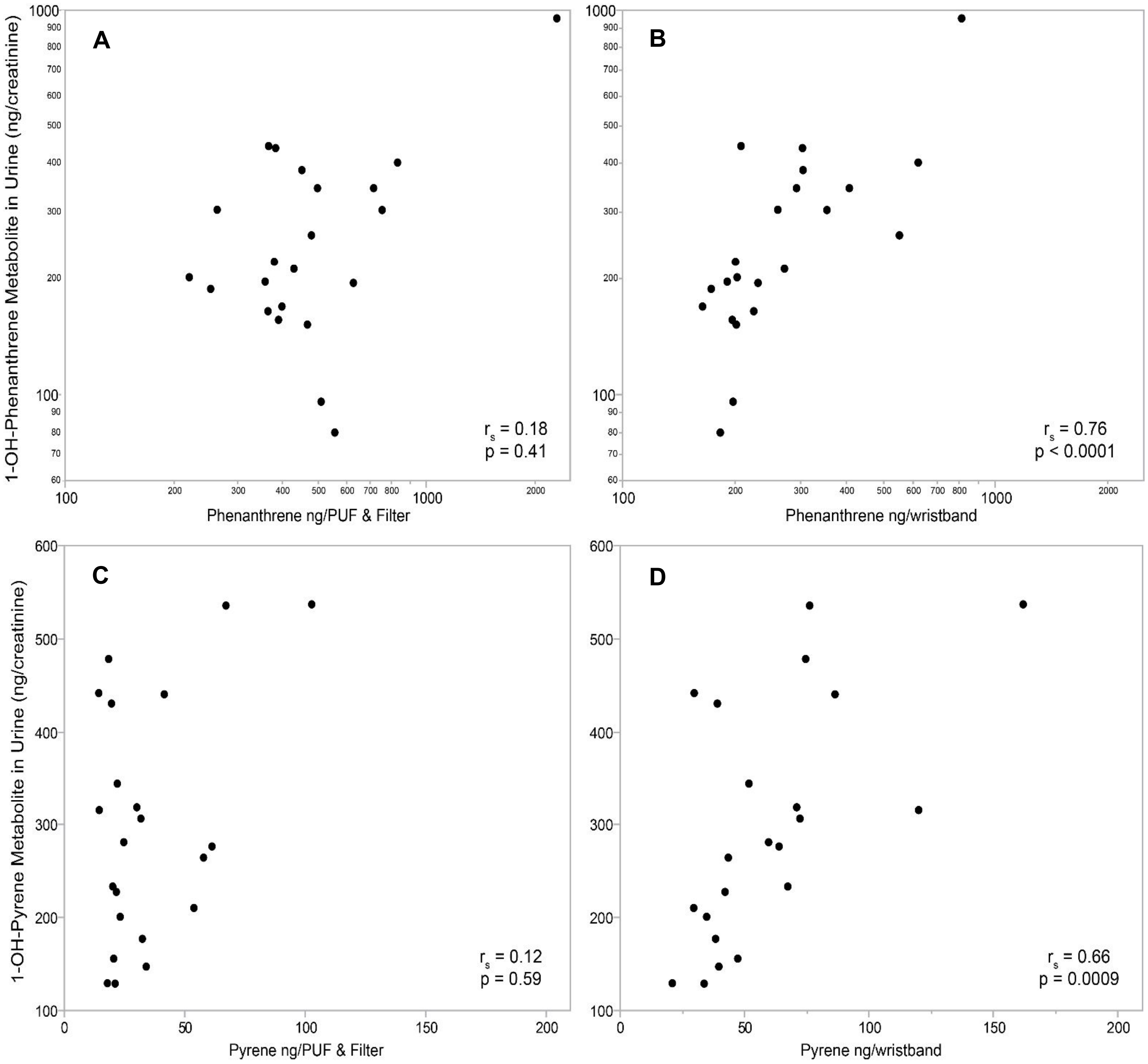

Fig. S1 Phenanthrene correlation scatterplots for OH-PAH concentrations in urine and PAH concentrations in A) PUFs and filters and B) wristbands. Pyrene correlation scatterplots for OH-PAH concentrations in urine and PAH concentrations in C) PUFs and filters and D) wristbands
